# Cost minimization analysis of line probe assay for detection of multidrug-resistant tuberculosis in Arkhangelsk region of Russian Federation

**DOI:** 10.1371/journal.pone.0211203

**Published:** 2019-01-29

**Authors:** E. N. Bogdanova, A. O. Mariandyshev, G. A. Balantcev, P. I. Eliseev, E. I. Nikishova, A. I. Gaida, D. Enarson, A. Detjen, R. Dacombe, P. P. J. Phillips, S. B. Squire, E. Gospodarevskaya

**Affiliations:** 1 Northern Arctic Federal University, Arkhangelsk, Russian Federation; 2 Northern State Medical University, Arkhangelsk, Russian Federation; 3 Arkhangelsk Clinical Antituberculosis Dispensary, Arkhangelsk, Russian Federation; 4 The International Union Against Tuberculosis and Lung Disease, Paris, France; 5 Liverpool School of Tropical Medicine, Liverpool, United Kingdom; 6 MRC Clinical Trials Unit at UCL, London, United Kingdom; 7 Deakin University, Melbourne, Australia; Harvard Medical School, UNITED STATES

## Abstract

**Background:**

The development of new diagnostic tools allows for faster detection of both tuberculosis (TB) and multidrug-resistant (MDR) TB and should lead to reduced transmission by earlier initiation of anti TB therapy. The research conducted in the Arkhangelsk region of the Russian Federation in 2012–14 included economic evaluation of Line Probe Assay (LPA) implementation in MDR-TB diagnostics compared to existing culture-based diagnostics of Löwenstein Jensen (LJ) and BacTAlert. Clinical superiority of LPA was demonstrated and results were reported elsewhere.

**Study aim:**

The PROVE-IT Russia study aimed to report the outcomes of the cost minimization analysis.

**Methods:**

Costs of LPA-based diagnostic algorithm (smear positive (SSm+) and for smear negative (SSm-) culture confirmed TB patients by Bactec MGIT or LJ were compared with conventional culture-based algorithm (LJ–for SSm- and SSm+ patients and BacTAlert–for SSm+ patients). Cost minimization analysis was conducted from the healthcare system, patient and societal perspectives and included the direct and indirect costs to the healthcare system (microscopy and drug susceptibility test (DST), hospitalization, medications obtained from electronic medical records) and non-hospital direct costs (patient’s travel cost, additional expenses associated with hospitalization, supplementary medicine and food) collected at the baseline and two subsequent interviews using the WHO-approved questionnaire.

**Results:**

Over the period of treatment the LPA-based diagnostic corresponded to lesser direct and indirect costs comparing to the alternative algorithms. For SSm+ LPA-based diagnostics resulted in the costs 4.5 times less (808.21 US$) than LJ (3593.81 US$) and 2.5 times less than BacTAlert liquid culture (2009.61 US$). For SSm- LPA in combination with Bactec MGIT (1480.75 US$) vs LJ (1785.83 US$) showed the highest cost minimization compared to LJ (2566.09 US$). One-way sensitivity analyses of the key parameters and threshold analyses were conducted and demonstrated that the results were robust to variations in the cost of hospitalization, medications and length of stay.

**Conclusion:**

From the perspective of Russian Federation healthcare system, TB diagnostic algorithms incorporating LPA method proved to be both more clinically effective and less expensive due to reduction in the number of hospital days to the correct MDR-TB diagnosis and treatment initiation. LPA diagnostics comparing conventional culture diagnostic algorithm MDR-TB was a cost minimizing strategy for both patients and healthcare system.

## Introduction

In 2016, four diagnostic tests were reviewed and recommended by WHO: the Xpert MTB/RIF, the loop-mediated isothermal amplification test for TB (TB-LAMP), two line probe assays (LPAs) for the detection of resistance to the first line anti-TB drugs isoniazid and rifampicin, and an LPA for the detection of resistance to second-line anti-TB drugs [1]. The introduction of new molecular genetic methods for diagnosis of *Mycobacterium tuberculosis* (*M*.*tb*) and drug resistance speeds up the multidrug and extensively drug resistant (MDR/XDR) diagnosis and makes it possible to start an appropriate treatment regimen sooner. The molecular genetic LPA was recommended by WHO as a rapid diagnostic tool to define drug susceptibility of *M*.*tb* in smear positive specimens or on isolates of specimens grown from smear negative specimens [2–4]. The LPA has a high accuracy for diagnosing both tuberculosis and multidrug-resistant tuberculosis [5]. However, there is insufficient data on the test’s clinical effectiveness in the context of the healthcare systems of different countries [6–10] and a paucity of evidence on its cost minimization in diagnostics and treatment of MDR-TB in the Russian Federation or the former Soviet republics [11–13]. Therefore, clinical effectiveness and cost minimization of LPA in comparison to the standard diagnostic tests (Löwenstein Jensen (LJ) solid culture, BacTAlert and BACTEC liquid cultures) in Russia warrants further investigation.

In 2009, under the USAID-funded TREAT TB initiative, Northern State Medical University in collaboration with the International Union Against Tuberculosis and Lung Disease, partners in South Africa, Brazil and at the Liverpool School of Tropical Medicine undertook the Policy Relevant Outcomes from Validating Evidence on Impact of LPA (PROVE IT LPA) study to be conducted in Arkhangelsk region (Russia) in 2012–14. The study included 5 layers of analysis (clinical effectiveness analysis, equity analysis, healthcare system, scale up analysis and policy analysis), and aimed to comprehensively assess the new TB diagnostic tests within the healthcare system context in different epidemiological settings and to define the measures needed to successfully implement new diagnostics within healthcare systems; health service perceptions of implementation of this new diagnostic tool have been analyzed [14].

Clinical studies have already proved the effectiveness of LPA as a rapid diagnostic tool to define drug susceptibility of *M*.*tb* in the Arkhangelsk region of the Russian Federation. The primary clinical outcomes were time to diagnosis of MDR-TB and treatment initiation. Treatment outcomes showed better results in LPA-based algorithm compared to culture-based algorithm in increasing treatment success rates among MDR-TB patients and decreasing in the number of patients who were lost to follow-up or died during treatment [15]. Mixed-method operational research with qualitative component study has demonstrated ways to facilitate the uptake of a diagnostic innovation. People take time to observe the effects of innovations, assess relative advantages and become convinced, sometimes by different types of evidence. Multidisciplinary opportunities for learning, reflecting on care pathways and adaptation should all be a part of introducing LPA diagnostics [16].

This paper covered the health economics component of LPA project by complementing results of the clinical trial (PROVE-IT Russia) [16] and a qualitative study [15] with cost minimization analysis of LPA implementation.

The study conducted in the Arkhangelsk region of the Russian Federation in 2012–14 included economic evaluation of LPA as a new diagnostic tool of MDR-TB in comparison to existing culture-based diagnostics (LJ and BacTAlert liquid cultures).

However, since the results of the clinical trial (PROVE-IT Russia) demonstrated clinical superiority of LPA as a new diagnostic tool of MDR-TB, the aim of this paper was to compare the cost of LPA versus the comparator diagnostic algorithms in Russia using a cost minimization analysis.

## Materials and methods

### Settings

#### General settings

The study was conducted in the civil population in the Arkhangelsk region, located in Northwest Russia. It is a 410 thousand square meters of the circumpolar surface area with a population of 1.13 million. There are 20 districts in the region with more than 50 hospitals and outpatient clinics [17].

#### TB management in the Arkhangelsk region

The specialized TB control services in the Arkhangelsk region consist of the regional antituberculosis dispensaries which have two separate inpatient departments for drug-sensitive and MDR-TB patients, outpatients department and outpatient TB cabinets in each districts General Hospitals. The Arkhangelsk clinical antituberculosis dispensary (ACAD) is a central facility performing diagnosis and treatment of tuberculosis in the region. Because of the high rates of MDR, all categories TB cases are tested for drug susceptibility at ACAD. SSm+ patients who are contagious are usually admitted to the in-patient department at ACAD. SSm- patients are managed at the district ambulatory TB units, but specimens are sent to ACAD for culture and DST.

### Ethics approval

The study was approved by the Ethics committee of Northern State Medical University, Arkhangelsk, Russian Federation on 4^th^ of June 2010 (approval protocol № 07/06) and the Ethics Advisory group of The Union on 5^th^ April 2011 (approval protocol № 01/11).

A waiver of informed consent was granted for the use of routine data. Additionally, informed consent was given by participants for their records to be used in this study for current cohort after 2011. All patient records information was anonymized and de-identified prior to analysis.

The CHEERS checklists [18] of the trial is listed in S1 File.

### Study population

All the patients diagnosed with MDR-TB in the Arkhangelsk region on the Russian Federation who were registered between September 2007 to August 2009 (163: 96 SSm+ and 67 SSm-) and from April 2011 to June 2012 (132: 60 SSm- and 72 SSm+ patients) were included in the study [15].

There was no statistically significant difference in socio-demographic and clinical characteristics of MDR-TB patients diagnosed with culture-based and LPA-based algorithms (Table 1).

**Table 1:** Characteristics of MDR-TB patients in the culture-based (old) and LPA-based (new) algorithms.

	Culture-based algorithm N = 163	LPA-based algorithm N = 132	Statistical analysis
Smear+ n (%)	96 (58.9%)	72 (54.5%)	Chi^2^ = 0.563, Df = 1, p = 0.453
Smear–n (%)	67 (41.1%)	60 (45.5%)	
Male (%)	132 (81%)	99 (75%)	Chi^2^ = 1.536, Df = 1, p = 0.215
Female (%)	31 (19%)	33 (25%)	
HIV-infected	3 (1.8%)	0 (0%)	Chi^2^ = 2.454, Df = 1, p = 0.117
Non-infected	160 (98.2%)	132 (100%)	
Average age, years	41.7±11.4	41.6±12.9	t-value– 0.071, Df = 293 p = 0.944
Average weight, kilo	61.7±11.5	60.3±10.5	t-value v 1.081, Df = 293, p = 0.281

Chi^2^ = Pearson Chi-squared test

### Sample and design

The primary clinical outcomes assessed in the clinical trial (PROVE-IT Russia) were: the time to correct diagnosis, and treatment initiation. Efficiency outcomes of implementation of LPA into TB diagnostic algorithms compared to the existing diagnostic algorithms are reported elsewhere [16].

Briefly, time to the treatment initiation, starting from the first sputum collection to the first full dose of anti-tuberculosis drugs, was statistically significantly shorter for SSm+ patients diagnosed by LPA (LPA-based algorithm), compared to patients diagnosed by BacTAlert or by LJ (culture-based algorithm). LPA, cultures and DST were performed according to national and manufacturer’s recommendations [19–22].

Similarly, statistically significant results were observed for the SSm- patients. The secondary clinical outcome was the success of treatment (i.e. the proportion of patients successfully treated less proportion of patients lost to follow up or dead). Overall treatment outcomes were better in LPA-based algorithm compared to culture-based algorithm (p = 0.003). The implementation of LPA was associated with an increase in treatment success rates among MDR-TB patients: 65.2% of patients diagnosed by LPA versus 44.8% patients diagnosed by LJ or BacTAlert. Accordingly, there was a decrease in the number of patients who were lost to follow-up or died during treatment [15].

The trial used the data on a historical cohort (September 2007-August 2009) as well as cross-sectional observations (April 2011-June 2012) [16].

**Fig 1:**
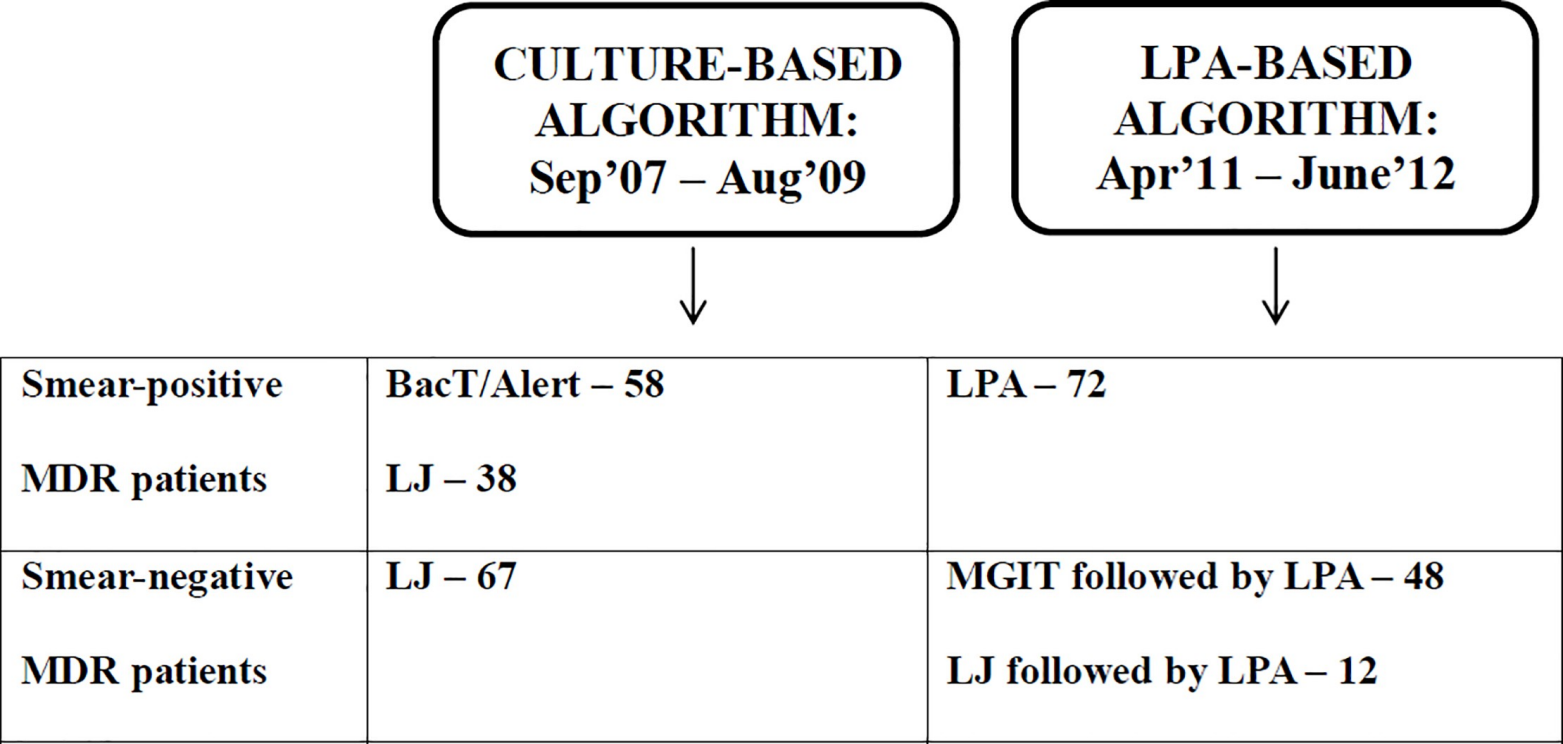
Study design, comparison of culture-based and LPA-based diagnostic algorithms for MDR TB used at ACAD between 2007 and 2012.

To assess cost minimization as a result of implementing LPA, the costs of diagnostics and treatment of the group diagnosed with either LJ or BacTAlert (i.e. under the ‘culture-based algorithm’ before LPA implementation) were compared with costs of the group diagnosed with either LPA or LPA in combination with LJ or Bactec MGIT (i.e. under a LPA-based algorithm after LPA was fully implemented) (Fig 1).

From September 2007 to August 2009, culture and DST were done by either BacTAlert for SSm+, or LJ for SSm- and also for SSm+ cases whenever BacTAlert results was unavailable due to no growth or contamination. Implementation of LPA for both first and second line DST started at ACAD in September 2009 (Hain Genotype MDRTB*plus* and MDRTB*sl* [23]).

During the period from April 2011 to June 2012, the new algorithm was fully implemented, with LPA replacing BacTAlert and LJ for SSm+ patients; in SSm- Bactec MGIT culture was performed first, followed by LPA on positive specimens. In cases where Bactec MGIT culture was unavailable due to no growth or contamination, LJ culture was followed by LPA.

### Cost minimization analysis

Cost minimization analysis was conducted from the healthcare system and societal perspectives, the latter including non-hospital direct costs. The time horizon for the economic evaluation was equal to the entire period of time taken for the MDR-TB treatment. ACAD’s accounting department determines the direct and indirect costs for MDR-TB diagnostics and treatment by using a full-cost model for assigning costs to each process. This model attributed all corresponding organizational costs to any process whose cost one intends to measure. The cost of the process included direct and indirect costs. Direct costs included the needs for medical material, personnel and diagnostic procedures. In this case cost of medical personnel was calculated including the staff working full time in ACAD. All personnel and indirect costs were obtained from the accounting department of ACAD.

#### Direct and indirect healthcare system costs

Healthcare system costs were assigned according to the accounting data specific to the universal healthcare coverage in Russia and included: cost of microscopy and drug susceptibility test (equipment, consumables and reagents), hospitalization, visits to other treatment units and medications.

Since prices of resource inputs vary considerably by country, we used, wherever available, the international prices for medications and laboratory supplies. The cost of MDR-TB drugs was covered by both federal and regional budgets of Ministry of Healthcare, therefore the listed prices, applicable to every state-funded medical facility in Russian Federation, were used. Standard international prices were not available for many laboratory supplies. In such instances, we used local prices of Arkhangelsk region (e.g. for calculating DST costs, cost of a hospital day, labour costs, patients’ expenses). Prices were converted to 2014 US$ with an exchange rate of 56.2584 rubles to US$1 as of 31.12.2014 [24].

- Costs of laboratory tests. All laboratory procedures (including bacteriological clinical laboratory tests) for both cohorts were broken down into their component parts and a detailed time and motion study was conducted. Depreciation of the equipment, consumables and reagents were included. We excluded expenses for the maintenance of property (building, transportation, auxiliary equipment, electricity and heating), because according to the incremental principle in cost data collection these costs are not changed when one DST test is substituted for another.
- Cost of hospitalization and visits to other treatment units. The cost of a hospital day in treatment varied depending on the treatment modality: SSm+ patients spent time as in-patients at ACAD until smear conversion, SSm- patients might receive treatment in “day care” (a patient came to the hospital every day, took medicines, got injections, consulted a doctor, stayed at the hospital for 3–7 hours and left for home), “hospital at home” (when medical staff brought medications to the patient’s residence) or as outpatient visit (ambulatory treatment). Costs of a hospital day and an outpatient visit were taken from the official federal medical documents [25]. Ministry of Healthcare in Russian Federation [26] provided an estimated unit cost for a medical facility in each category: cost per “bed-day” (for an in-patient department), cost per a “patient-day” (for “day care”) and cost per “visit” (for ambulatory care and “hospital at home”). These federal standards were calculated for all TB patients but the costs of MDR-TB diagnostics and treatment (primarily costs of DST and medications) were much higher than it was shown in those federal standards. Therefore, we calculated cost of hospitalization and medications using official documents of ACAD to assess real costs of treatment and diagnostics for MDR-TB patients.
- Cost of MDR-TB medications and other pharmaceuticals administered to the patients. Prices for medications were obtained from the list of prices for the drugs included in the list of vital and essential medicines approved by the agency on tariffs and prices for the Arkhangelsk region on August, 31 in 2012 [27, 28].

To calculate the total cost of healthcare resources, unit costs for DST and other tests, medications, hospital and outpatient visits were applied to each patient depending on their individual drug regimen and treatment modalities utilized over all the period of diagnostics and treatment. Individual clinical data (number of days in treatment by each treatment modality, medication regimens, number and type of laboratory tests) were obtained from ACAD electronic recording and reporting system (INIT-TB).

#### Non-hospital direct costs

For the LPA-based new algorithm patient costs were collected at the baseline and at two subsequent interviews (roughly at 3 and 6 months after enrollment) using the questionnaire developed by WHO [29] and adapted to the Russian socioeconomic context. The costs included travel cost incurred by both patients and guardians (relatives who accompany patients to appointments), additional expenses associated with hospitalization, supplementary medicine and food. The same unit costs were applied retrospectively to the control group of MDR-TB patients who were treated under the culture-based algorithm. Although the absence of the actual patient costs incurred prior to LPA implementation might introduce a bias in the results, it still allowed to estimate the difference in the societal cost of MDR-TB diagnosis and treatment.

Mean costs per unit of resources used (per hospital day, per test etc.) were calculated from the quantity and unit prices of resources. These derived costs per unit of resources are presented in Table 2 that shows the dollar value for each of the cost components for each cohort of patients.

**Table 2:** Costs, including diagnostic and treatment for LPA- and culture-based algorithms.

№	Unit costs	*CULTURE-BASED ALGORITHM*			*LPA-BASED* *ALGORITHM*		
		BacTAlert (SSm+)	LJ (SSm+)	LJ (SSm-)	LPA (SSm+)	LPA+ Bactec MGIT (SSm-)	LPA+LJ (SSm-)
1	DIRECT AND INDIRECT HEALTHCARE SYSTEM COSTS						
1.1	Hospitalization (“bed day” or “patient day” or “visit”), per 1 day, US$	10.09	10.08	9.28	10.90	8.64	8.41
1.2	Medications, mean per 1 day, US$	11.42	11.02	10.36	12.73	11.36	11.36
1.3	Microscopy and drug susceptibility test, per 1 test (consumables and reagents, depreciation of the equipment per 1 DST), US$	181.48	4.96	4.96	26.25	137.07	31.21
1.4	Other laboratory tests (blood, urine etc.), per 1 day, US$	0.02	0.02	0.02	0.02	0.02	0.02
2	NON-HOSPITAL DIRECT COSTS						
	per 1 day, US$	0.71	0.71	0.78	0.71	0.78	0.78

### Sensitivity analysis

Sensitivity analysis determined the level of uncertainty associated with variation around the observed parameters of costs and outcomes of clinical interventions. A one-way sensitivity analysis varied one cost parameter at a time to estimate its impact on the results of cost minimization analysis. We performed a variation of a one-way sensitivity analysis (a threshold analysis) to assess the robustness of the base case results to changes in cost values.

### Analysis and statistics

All data were obtained from official federal medical documents, official accounting and medical documents and an electronic recording and reporting system called INIT-TB, which has been used in ACAD since 2007. Double data entry was used for all information. Statistical analyses were performed using Microsoft Excel 2010, Mathworks MATLAB 2009 and STATISTICA 6.0 by StatSoft Inc.

## Results

The costs associated with MDR-TB diagnostics and treatment of all the patients diagnosed MDR-TB in the Arkhangelsk region on the Russian Federation who were registered between September 2007 to August 2009 (163: 96 SSm+ and 67 SSm-) and from April 2011 to June 2012 (132: 60 SSm- and 72 SSm+ patients) were estimated (Table 1).

For SSm- LJ had the lowest mean unit costs per medications (10.36 US$), microscopy and drug susceptibility test (4.96 US$) while this cost for LPA in combination with Bactec MGIT was 13 times higher (137.07 US$). LPA diagnostics in combination with LJ was associated with less hospitalization costs (8.41 US$). For SSm+ LJ also had the least unit costs of hospitalization (10.08 US$), medications (11.02 US$) and microscopy and drug susceptibility test (4.96 US$), while LPA diagnostics resulted in higher unit costs of hospitalization (10.90 US$) and medications (12.73 US$). BacTAlert liquid culture diagnostics had the most expensive microscopy and drug susceptibility test (181.48 US$) (Table 2).

However, analysis of resource-utilization as average per stakeholders (healthcare system, patients and society) proved that LPA-based diagnostic algorithm for both SSm+ and SSm- resulted in the least costs. For SSm+ average direct and indirect healthcare system costs and non-hospital direct costs per patient were: LPA– 785.42 US$ and 22.79 US$, BacTAlert liquid culture– 1951.25 US$ and 58.36 US$, LJ– 3477.09 US$ and 116.72 US$. It was a result of faster LPA diagnostics– 32.1 days to MDR-TB diagnosis compared to BacTAlert liquid culture (82.2) and LJ (164.4). For SSm+ average direct and indirect healthcare system costs and non-hospital direct costs per patient were: LPA in combination with Bactec MGIT– 1430.36 US$ and 50.39 US$, LPA in combination with LJ– 1719.30 US$ and 66.53 US$.

It proved that the LPA-based diagnostic and treatment algorithm was associated with lesser costs comparing to the alternative. For SSm+ LPA-based diagnostics resulted in the societal costs per patient 4.5 times less (808.21 US$) than LJ (3593.81 US$) and 2.5 times less than BacTAlert liquid culture (2009.61 US$). For SSm- LPA in combination with Bactec MGIT (1480.75 US$) vs LJ (1785.83 US$) showed the highest cost minimization compared to LJ (2566.09 US$) (Table 3).

**Table 3:** Resource-utilization for LPA- and culture-based algorithms.

Diagnostic strategy: LPA vs Comparator	Comparators	Average number of days to the MDR-TB diagnosis	Average direct and indirect healthcare system costs per patient (US$)	Average non-hospital direct costs per patient (US$)	Average societal costs per patient (US$)
LPA vs BacTAlert liquid culture (SSm+)	LPA	32.1	785.42	22.79	808.21
	BacTAlert	82.2	1951.25	58.36	2009.61
LPA vs LJ (SSm+)	LPA	32.1	785.42	22.79	808.21
	LJ	164.4	3477.09	116.72	3593.81
LPA+Bactec MGIT vs LJ (SSm-)	LPA+Bactec MGIT	64.6	1430.36	50.39	1480.75
	LJ	125.3	2468.36	97.73	2566.09
LPA+LJ vs LJ (SSm-)	LPA+LJ	85.3	1719.30	66.53	1785.83
	LJ	125.3	2468.36	97.73	2566.09

The results of sensitivity analysis were fairly robust to the variations in the cost drivers. In particular, even significant (doubling) change in the cost of a LPA-based diagnostic method (equipment and laboratory costs) hardly affected the results indicating that investing in LPA was a cost minimization strategy for the healthcare system.

## Discussion

Tuberculosis causes high rates of mortality and high economic costs on the society in many low-income countries. Especially MDR/XDR-TB is a great burden because of huge costs of second-line medications, administrative resources, etc. Multiple studies about TB burden sustained the fact that costs of TB treatment varied in different countries due to level of country development, national features, cultural traditions and running household [11, 30–33]. Consequently, the implementation of new diagnostic tools to provide fast and accurate diagnosis can increase cost-effectiveness of TB treatment and get positive impact on quality of medical service.

For a long time, culture-based methods remained the gold standard for TB diagnostics and they are currently reference standard for drug susceptibility testing. But globally, the use of rapid molecular tests is increasing: Xpert MTB/RIF, rapid line probe assays (LPAs), a rapid LPA that tests for resistance to fluoroquinolones and injectable anti-TB drugs (referred to as a second-line LPA) and sequencing technologies. First-line LPAs were first recommended by WHO in 2008; the second-line LPA was first recommended in May 2016 [1]. However, some research still show that the LPA cannot completely replace phenotypic culture methods [34]. High costs of diagnostic equipment, its maintenance and consumables need extra investments and make it extremely important to assess cost minimization of innovative methods for TB diagnostics based on full societal costs (including overall health service-related and patient’s cost).

We presented an economic evaluation of the implementation of LPA as a molecular-genetic method for MDR-TB diagnostics compared to culture-based diagnostics (LJ and BacTAlert liquid culture). Clinical outcomes of LPA implementation in Russia proved its high effectiveness [15]. These findings were line with other recent studies that Line Probe Assay is a rapid tool for screening TB and DST [7, 8, 10, 15, 34–41]. The economic evaluation of LPA proved that it was a cost minimizing strategy for both Healthcare system (with cost minimization observed in costs of hospital days, laboratory costs, medications) and for patients.

The cost of hospitalization and visits to other treatment modalities varied in different countries because of salary rates of the medical staff, administrative costs, etc. For example, in southern African countries (Bostwana, Lesotho, Namibia, South Africa and Swaziland) cost of outpatient diagnostic visit, outpatient treatment visit and inpatient care varied considerably (prices in US$2012): 2.94–10.32, 1.95–6.85 and 8.78–38.99, respectively [42]. In sub-Saharan Africa mean cost of health service visit per patient was 8.27–10.46 (prices in US$2014) [43]. In the following research basic value of outpatient visit (diagnosis or follow up) was estimated as US$10 [44, 45]. In Brazil cost of follow-up medical visit (n = 3) was 2.51–43.87 [46], hospital room costs– 4.16 per day; costs of clinical staff salary and clinical consultations– 2.52 per patient; and costs of clinical nursing consultations– 2.52 per patient (prices in US$2014) [47].

In the Arkhangelsk region of Russia, in 2014 costs of hospitalization and visits to other treatment units were even less than in African countries –1.67–16.57 US$, standard outpatient visit was 7.5 US$. Hospital-based services costs for SSm+ patients diagnosed with LPA-based algorithm were 10.90 US$ per day, which was higher than the corresponding cost for BacTAlert of 10.09 US$ and LJ-based algorithm (10.08 US$); for SSm- patients diagnosed with LPA combined with Bactec MGIT the cost was 8.64 US$ per day and for those diagnosed with combination of LPA and LJ the cost was 8.41 US$; both comparing favorably with cost per day associated with the LJ-based algorithm (9.28 US$).

Costs for microscopy and DST is a principal parameter which has great influence on cost of a diagnostic test. The WHO recommends their prices and budgeting: i.e. annual budget including costs of equipment, maintenance, consumables, human resource, installation and running costs. But real cost of a test differs worldwide and depends on infrastructure facilities, and organization of healthcare system in different countries etc. In our study, we evaluated costs of LJ, Bactec MGIT, BacTAlert and LPA. Comparison with costs of TB diagnostic tests in other countries showed that these values in Russia were comparable. In 2010–14 costs of culture-based diagnostic tests in different countries worldwide varied 12.35–15.45 US$ (culture–LJ), 10.51–52.60 US$ (culture–MGIT), 22.33–23.98 US$ (DST–LJ), 15–232.00 US$ (culture DST–MGIT), 33.01–38.82 US$ (DST–MGIT+LPA), 1.36–23.07 US$ (DST–LPA on sputum) [42, 43, 45–53].

In our study costs for microscopy and DST also varied considerably–from 4.96 US$ (LJ) to 181.48 US$ (BacTAlert); resulting in LJ being the least expensive diagnostic tool, followed by LPA, while BacTAlert and the combination of LPA and Bactec MGIT were the most expensive DST methods. Medical supplies (i.e., consumables and reagents) were the major determinant of costs for LPA and LJ tests, but up to 88 samples could be produced from one kit. While depreciation of the equipment was rather low. In comparison, BacTAlert was an expensive DST because of the high cost of equipment and medical supplies per 1 test. Compared to the cheaper LPA and LJ, BacTAlert was a less attractive diagnostic alternative.

MDR-TB treatment was extremely expensive due to high costs of medications. Their prices varied significantly in different countries which causes a huge range of cost-effectiveness estimations for new TB diagnostic tools. For example, in India, the cost of second-line standard treatment regimen for 24 months was 4,204–7,421 [50], in Uganda– 1000.00–5000.00 [52] (prices in US$2013), in sub-Saharan Africa monthly MDR-TB regimen cost was 119.37–179.06 [42], in South Africa MDR-TB treatment per day– 2.71–29.52 [43], in Brazil– 120.63–691.24 [46] (prices in US$2014). In our study over the whole course of treatment average expenses for MDR-TB medications per patient to the healthcare system were 7369.5 US$. The mean costs of all medications per day varied between the cohorts from 10.36 US$ (LJ for SSm-) to 12.73 US$ (for LPA SSm+). These mean costs estimates included 0.6 US$ for the first line TB medications (H, R, Z, E) per day. The economic evaluation of LPA using the primary clinical outcome of the difference in time from collection of the first sputum specimen to the start of MDR-TB treatment presented a considerable challenge. Over the short time horizon, the cost per LPA-diagnosed patient who started on the more expensive MDR-TB drugs earlier would largely exceed the cost per patient who still continued receiving first line TB treatment while awaiting the correct diagnosis with an alternative diagnostic method. The immediate increase in cost of MDR-TB medications was offset by reduction in hospital costs for an average SSm+ patient. There was no equivalent cost reduction in SSm- patients who received ambulatory treatment before and after MDR-TB diagnosis. The reduction in cost in SSm- patients was due to discharging from the hospital. Therefore, LPA-based algorithm provided cost minimization of health service-related costs for both SSm+ and SSm- patients.

Patient and their guardians (accompanying persons) incured some expenses due to the additional expenses associated with hospitalization, supplementary medicine and food. LPA-based diagnosis algorithm corresponded to the speedier assignment of the correct treatment (by mean value of 50.1 days in comparison to SSm+ patients diagnosed with BacTAlert, and by 132.3 days in comparison to SSm- patients diagnosed with LJ). The speedier diagnosis resulted in the earlier discharge of SSm+ patients from hospital, who then faced higher daily travel expenditures to collect drugs from a medical facility. In our study, most of TB patients belonged to the socially disadvantaged groups and were entitled to the partially or fully subsidized services such as public transport (travelling to medical facilities for free) or supplementary medications (provided at a large discount). Our results reflected these social arrangements showing a daily average out-of-pocket expenses of 0.71 and 0.78 US$ for SSm+ and SSm- patients respectively, which was equivalent to and showed that patient costs were considerably smaller than in other countries without similar safety net.

Molecular genetic methods for TB diagnostics proved cost minimization in different research [37, 45]. Though there is insufficient data of evaluation of cost minimization associated with implementation of LPA-based algorithm. In our study LPA-based algorithm showed large cost minimization for the Russian Healthcare system ranging from 780.26 to 2785.60 US$.

Our study had several limitations. The before and after design was associated with an inherent risk of misattributing the cost reduction to the change in diagnostic practices and overlooking the accumulated effects of other changes in provision of TB treatment. There was only a limited number of characteristics between the intervention and the control group of patients that was tested for the statistical significance, so the systematic causes of heterogeneity could not be ruled out. The prices for medications varied strongly during the study period because they were imported by different pharmaceutical companies. Medical products bought and received by ACAD in many lots and their prices differed considerably. It was impossible to identify what lot of medications was used for curing the group of MDR-TB patients. To solve this issue limited prices recommended by Federal Service on Surveillance in healthcare (Roszdravnadzor) [54] were implemented for calculations. It provided neutralizion of inflation factors but moved to possible inaccuracy of assessment of actual cost of MDR-TB drugs. Since no data on the actual patient costs in the control group were available, the reduction was attributed to that possibility that patients faced additional transport costs after earlier discharge from the hospital under LPA and, therefore the results should be interpreted with caution. In our study costs associated with LPA and cultural DST were calculated as if every patient was confirmed with MDR after initial test and there were no erroneous or indeterminate results. Concordance of LPA and cultural DST was high as published in multiple studies including a study performed earlier in Arkhangelsk region. At the same time, it was possible that errors could occur while performing different tests for drug resistance resulting not only in wrong diagnosis and treatment but also in increased health care costs thus laboratory quality control was an important issue that should be evaluated.

The study conclusions may be equally valid in other regions of the Russian Federation, because of the standard MDR-TB treatment practices set by the Federal regulations. Although the prices of MDR-TB medication and labor costs can differ considerably in various parts of Russia, our results were robust to variations in costs of medications and costs per bed day. Genetic molecular methods were recommended by the Ministry of Healthcare but regional health providers are still using different diagnostic tools to test TB and MDR-TB (solid and liquid mediums in parallel with GeneXpert, country molecular genetic tests: Biochip 2, Synthol, etc.). Patient costs (as well as household costs) may differ in other regions because of specific socioeconomic conditions, geographical position of the territory and regional variations in public health support for TB patients. Arkhangelsk regional TB service is involved in effective international cooperation due to medical research activity and clinical trials, which help to realize social initiatives: i.e., free additional food for TB patients, modern equipment for TB diagnostics, new medications etc.

The benefits of LPA implementation received in our study (reduced time to correct diagnosis and treatment initiation and cost minimization) can have positive effects on the dynamics of the testing/treatment strategy in the other regions of the Russian Federation.

## Conclusions

Implementation of the LPA in Arkhangelsk region of the Russian Federation was associated with both reduction of time to correct MDR-TB diagnosis and cost minimization to the health care system. Patients diagnosed with LPA also avoided some costs incurred by patients diagnosed by conventional methods. The most significant reduction of time to diagnosis and treatment initiation of MDR-TB and the associated cost minimization were observed in the subgroup of sputum positive patients.

Clinically superior LPA-based algorithm was associated with cost reduction to Russian healthcare system, and total cost minimization in treatment-related expenditures to patients most of whom belong to the low-income subgroup. Our comparative economic analysis of MDR-TB diagnostics and treatment algorithms suggested that a targeted subsidy/home treatment might provide an incentive for MDR-TB patients to stay in treatment and lessen the burden of disease.

The LPA-based diagnostic and treatment algorithms were more effective in delivering the correct diagnosis sooner and are also less expensive than the alternatives. Therefore, the LPA was a dominant intervention.

PROVE IT was supported *by a United States Agency for International Development (USAID) Cooperative Agreement (TREAT TB–Agreement No*. *GHN*‐*A*‐*00*‐*08*‐*00004*‐*00)*. The contents are the responsibility of the authors and do not necessarily reflect the views of USAID.

We have no conflict of interest to declare.

## Supporting information

S1 File CHEERS checklist. (PDF)
